# A prognostic signature based on three non‐coding RNAs for prediction of the overall survival of glioma patients

**DOI:** 10.1002/2211-5463.12602

**Published:** 2019-03-07

**Authors:** Junmin Xian, Quanzhong Zhang, Xiwen Guo, Xiankun Liang, Xinhua Liu, Yugong Feng

**Affiliations:** ^1^ Department of Neurosurgery The Affiliated Hospital of Qingdao University China; ^2^ Department of Neurosurgery Heze Municipal Hospital China; ^3^ School of Biomedical Engineering Tianjin Medical University China

**Keywords:** GEO, glioma, GSEA, non‐coding RNA, prognostic signature, random survival forest

## Abstract

Recent studies have identified certain non‐coding RNAs (ncRNAs) as biomarkers of disease progression. Glioma is the most common primary intracranial cancer, with high mortality. Here, we developed a prognostic signature for prediction of overall survival (OS) of glioma patients by analyzing ncRNA expression profiles. We downloaded gene expression profiles of glioma patients along with their clinical information from the Gene Expression Omnibus and extracted ncRNA expression profiles via a microarray annotation file. Correlations between ncRNAs and glioma patients’ OS were first evaluated through univariate Cox regression analysis and a permutation test, followed by random survival forest analysis for further screening of valuable ncRNA signatures. Prognostic signatures could be established as a risk score formula by including ncRNA signature expression values weighted by their estimated regression coefficients. Patients could be divided into high risk and low risk subgroups by using the median risk score as cutoff. As a result, glioma patients with a high risk score tended to have shorter OS than those with low risk scores, which was confirmed by analyzing another set of glioma patients in an independent dataset. Additionally, gene set enrichment analysis showed significant enrichment of cancer development‐related biological processes and pathways. Our study may provide further insights into the evaluation of glioma patients’ prognosis.

**Abbreviations**
CNS: central nervous system
EGFR: epidermal growth factor receptor
GEO: Gene Expression Omnibus
GO: gene ontology
GSEA: gene set enrichment analysis
HR: hazard ratio
KEGG: Kyoto Encyclopedia of Genes and Genomes
MGMT: *O* ^6^‐methylguanin‐DNA methyltransferase
ncRNA: non‐coding RNA
OS: overall survival
PON2: paraoxonase 2
qRT‐PCR: quantitative real‐time PCR
USP46: ubiquitin specific peptidase 46

Glioma is the most prevalent primary brain malignant tumor. The tumor originates from the glial cells and develops into a heterogeneous tumor in the central nervous system (CNS) 1, 2. Gliomas can be divided into four different grades according to their severity defined by the World Health Organization, with grades I and II, pilocytic astrocytoma and diffuse astrocytoma, constituting low‐grade gliomas, and grades III and IV, anaplastic astrocytoma and glioblastoma, constituting high‐grade gliomas 3, 4. The occurrence rate of gliomas was 6–7 cases per 100 000 worldwide, and 60% of gliomas in adults were anaplastic astrocytoma and glioblastoma malignant tumors. The average overall survival (OS) of these patients was less than 15–20 months 5, 6, 7. Although significant advances have been achieved in diagnostic and therapeutic methods, the elevated incidence of glioma requires that more attention be paid to early diagnosis and prognosis supervision 6. To improve the diagnostic accuracy and meaningful survival, histopathological features of glioma patients can be investigated to illustrate glioma pathogenesis.

As an authentic reflection of body characteristics, biomarkers can be measured easily and their changes *in vivo* can provide disease information for early development and prognosis and even the grades and subtypes differentiating cancers 8, 9, 10, 11, 12. Some glioma biomarkers have been found to have significant functions. Genome‐wide sequencing in glioblastoma patients revealed a mutation of the *IDH* gene that was more prevalent in patients with secondary glioblastoma, and which can be regarded as a potential biomarker to predict glioblastoma development 13, 14. Adachi‐Hayama *et al*. 15 reported that the level of anti‐filamin C antibody in low‐grade glioma was significantly higher than in high‐grade glioma patients and healthy control, suggesting that the anti‐filamin C antibody could be regarded as a potential serum biomarker for the early diagnosis of different grades of glioma. Circulating microRNAs were also studied for their potential diagnostic role for glioma. Aberrant serum miR‐451 levels in glioma patients were observed, and down‐regulation of miR‐451 was tightly associated with a lower survival rate in glioblastoma patients, suggested that miR‐451 could act as a potential circulating indicator for the prognosis of glioma patients 16.

Considering the complex heterogeneous nature and transitivity of cancer, a single biomarker is not enough, owing to insufficient sensitivity and specificity, for early diagnosis and accurate prediction of prognosis, and additional biomarkers should therefore be further explored. Non‐coding RNAs (ncRNAs) are a type of functional RNA without a protein‐coding role that can regulate biochemical processes *in vivo* 17. Accumulated evidence has shown that ncRNAs play an important role in cancer development, progression and metastasis 18, 19, 20, 21, and they have been proposed as promising diagnostic and prognostic biomarkers in cancer 22, 23, 24, 25, 26. It has been revealed that the abnormal expression of ncRNA appears to be involved in CNS cancers such as glioblastoma tumors, and such ncRNAs include *HOTAIR* 27, *MALAT1* 28 and *HIF1A‐AS2* 29. Although an immune‐related long non‐coding RNA has been used as an independent prognostic marker 30, the clinical implication of ncRNA signatures for glioma patients requires further research.

In this study, genome‐wide expression profiles based on a gene microarray of 80 glioma samples were obtained from the Gene Expression Omnibus (GEO), and the expression profiles of ncRNAs were extracted and analyzed for their associations with glioma OS. Besides this, random survival forest analysis identified a prognostic signature that is the weighted composition of expression values of three ncRNAs, namely *LOC441179*,* PON2* and *USP46‐AS1*, that could accurately separate glioma samples with longer OS from those with shorter OS. This study should be helpful for prediction of glioma prognosis of specific patients and selection of a suitable therapeutic method.

## Materials and methods

### Study population

Glioma patients’ gene expression profiles along with their clinical information in this study were obtained from the GEO. https://www.ncbi.nlm.nih.gov/geo/query/acc.cgi?acc=GSE7696 31, which contains 80 glioma samples and four normal controls, was used as the training set for assessment of associations between ncRNA and patients’ OS. Further, we downloaded another dataset, https://www.ncbi.nlm.nih.gov/geo/query/acc.cgi?acc=GSE43378 32, which is composed of 50 glioma samples, as the validation set. Both the datasets were profiled based on the Affymetrix HG‐U133 Plus 2.0 Array (Affymetrix, Waltham, MA USA).

### Dataset preprocessing

Raw CEL files were imported into r programming software, and background correction and normalization were performed by using the affy bioconductor package 33. Only probes that were annotated as ‘non‐coding’ were retained, and average expression values were adopted for ncRNAs that were annotated by multi probes, which generated a total of 10 641 probes that annotated 8567 ncRNAs.

### Gene set enrichment analysis

Gene set enrichment analysis (GSEA) was performed by using gsea version 3 software 34. We used ‘C5.All.v6.1.symbols.gmt’ and ‘C2.CP.v6.1.symbols.gmt’ as gene sets, which represent canonical gene ontology (GO) terms and Kyoto Encyclopedia of Genes and Genomes (KEGG) pathways, respectively. The numbers of permutation was set to 1000, and permutation *P* < 0.05 was considered as statistically significant. The EnrichmentMap plug‐in of cytoscape software 35, 36 was then applied for visualization of significantly enriched functions.

### RNA extraction and qRT‐PCR

Quantitative real‐time PCR (qRT‐PCR) was used to examine the expression of *LOC441179*,* PON2* and *USP46‐AS1* in glioma cells. Total RNAs were extracted from the glioma cells and control cells with TRIzol reagent (TaKaRa, Dalian, China) according to the manufacturer's instructions. cDNA synthesis was performed with PrimeScript™ RT reagent Kit with gDNA Eraser (TaKaRa) according to the manufacturer's instructions. The primers specific of each ncRNA were designed using the primer premier 5.0 program (PREMIER Biosoft, Palo Alto, CA, USA) according to the sequences obtained from NCBI (Table 1). The β‐actin gene was chosen as the reference for internal standardization. The qRT‐PCR amplification was performed on an ABI 7500 real‐time PCR system (Applied Biosystems, Waltham, MA, USA) at 95 °C for 10 s, followed by 40 cycles of 95 °C for 5 s, 60 °C for 15 s, 72 °C for 35 s. Reaction of each sample was performed in triplicate. At the end of each reaction, dissociation analysis was performed to confirm the amplification specificity. The expression levels of *LOC441179*,* PON2* and *USP46‐AS1* relative to that of the β‐actin gene in glioma cells and control cells were calculated by the comparative *C*_T_ method ($2^{-\Delta \Delta C_{T}}$).

**Table 1:** Primers used for real‐time PCR analysis

LncRNAs	Primers (5′–3′)	Amplicon size (bp)
*LOC441179*	5′‐GCATAGCCCTACTTCTCCAAACCAC‐3′	179
	5′‐TGTTCTCATTTCTTCTTTTGACCTC‐3′	
*PON2*	5′‐TCCAAATGAAGTTAAAGTGGTAGCA‐3′	169
	5′‐ATCCAGCTCAAGTACCTTCAACTGA‐3′	
*USP46‐AS1*	5′‐CATTTGATTCCCTGCCTCTTTCTAT‐3′	214
	5′‐AACATTTCGGTAAGTCATCTGGGCA‐3′	

### Statistical analysis

Associations between ncRNAs and glioma patients’ OS were assessed by using univariate Cox regression analysis along with a permutation test. The RSF method was then applied for further screening of valuable ncRNA signatures with the randomforest R package. A prognostic signature could be established by including valuable ncRNAs’ expression values weighted by their multivariate Cox regression coefficients. Patients were divided into low risk and high risk subgroups according to the median risk score that was obtained based on the prognostic signature. The Kaplan–Meier method along with the log‐rank test was adopted for evaluation of differences in OS between patients with low risk and high risk score. All of the statistical analysis was conducted through r version 3.4.3 with *P* < 0.05 considered as statistically significant.

## Results

### ncRNA signatures

**Figure 1:**
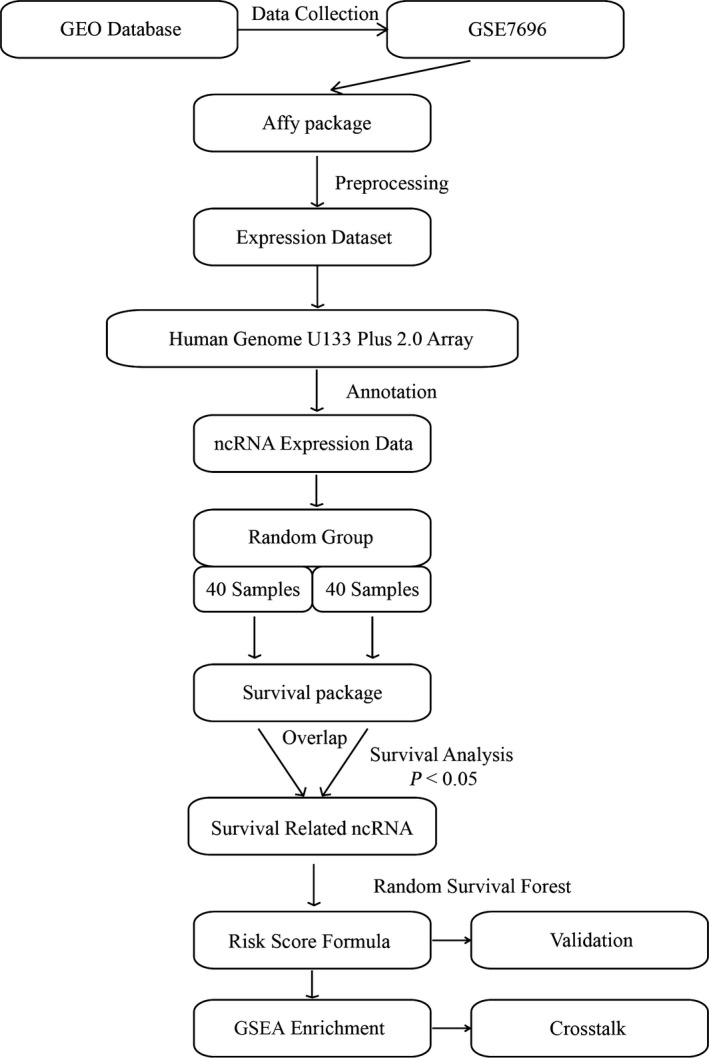
Workflow of the study.

**Figure 2:**
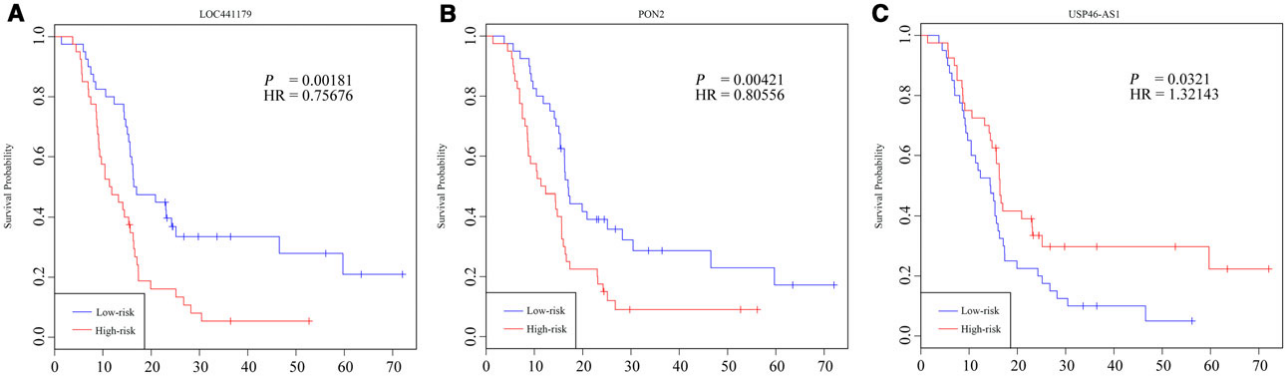
Kaplan–Meier curve analysis of glioma patients’ OS stratified by the expression values of *LOC441179*,*PON2* and *USP46‐AS1*.

Figure 1 illustrates the workflow for identification and evaluation of a prognostic signature. As shown, glioma patients in the training set, i.e. https://www.ncbi.nlm.nih.gov/geo/query/acc.cgi?acc=GSE7696, were randomly divided into two groups, and both of them were used for identifying ncRNAs significantly associated with glioma OS. As a result, a total of 374 and 519 ncRNAs were found to be significantly related to glioma OS by univariate Cox regression analysis in the two groups. We found 24 overlaps between the two lists of ncRNAs, and three ncRNA signatures, namely *USP46‐AS1*,* PON2* and *LOC441179*, as shown in Table 2, remained after subjecting the 24 overlaps to the random survival forest model and were used for the establishment of a prognostic signature. Of those three ncRNAs, a higher expression value of *LOC441179* (Fig. 2A) and *PON2* (Fig. 2B) indicated shorter glioma OS, which corresponded to their positive estimated coefficients, while patients with a higher *USP46‐AS1* (Fig. 2C) expression value tended to have longer OS, which was consistent with its negative estimated coefficient.

**Table 2:** Gene signatures obtained through RSF method. CI, confidence interval

Gene symbol	Alignment	Hazard ratio	95% CI	*P*
*USP46‐AS1*	chr4:53527057–53527665	1.32	0.0022–0.8097	0.0321
*PON2*	chr4:1160722–1166597	0.81	0.3001–1.5439	0.0042
*LOC441179*	chr6:168198474–168198927	0.76	0.9303–2.4619	0.0018

### Prognostic signature

**Figure 3:**
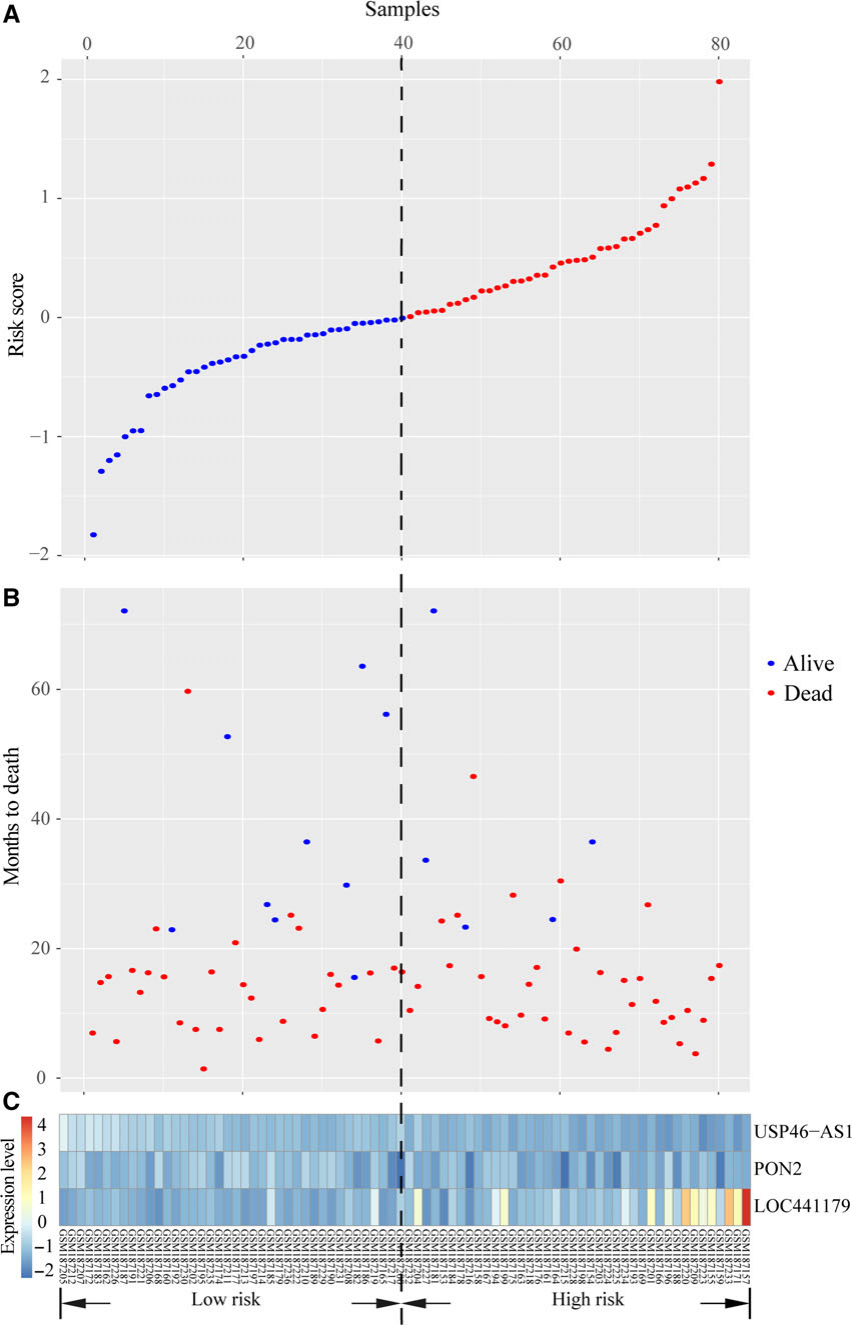
Distribution of risk score, OS and expression profiles of the three ncRNA signatures in the training set. (A) Distribution of glioma patients’ risk score. (B) Distribution of OS time and status of glioma patients. (C) Heatmap representing expression profiles of the three ncRNA signatures in glioma patient samples with rows and columns representing ncRNA and samples, respectively. Black dashed line indicates the median risk score.

We established the prognostic signature as a risk score formula by subjecting the three ncRNA expression values to multivariate Cox regression analysis in the training set as follows: risk score = −3.1563 × expression level of *USP46‐AS1* + 0.3846 × expression level of *PON2* + 0.4144 × expression level of *LOC441179*. The risk score of samples in the training set was calculated and ranked in ascending order. Figure 3A shows the distribution of the risk score. Figure 3B illustrated that patients with lower risk score have better OS than those with higher risk score. Expression values of both *PON2* and *LOC441179* were higher in patients with high risk score than in those with low risk score, while patients with high risk score tended to express a low *USP46‐AS1* level (Fig. 3C).

### Stratification analysis

**Figure 4:**
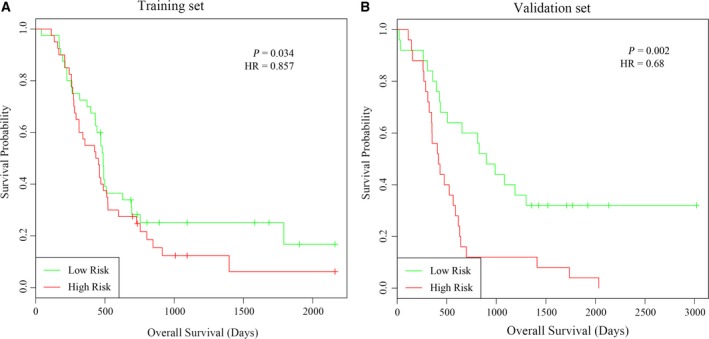
Kaplan–Meier curve analysis of glioma patients’ OS stratified by risk score in the training and validation sets.

Glioma patients in the training set as well as in the validation set were classified into high‐risk and low‐risk subgroups by using their median risk scores as cutoff. We used the Kaplan–Meier method along with the log‐rank test to assess differences in OS between high and low risk score. As a result, patients with a high risk score tend to have shorter OS time than those with a low risk score in the training set as shown in Fig. 4A. Additionally, analysis of glioma patients in the validation set also confirmed this result (Fig. 4B), which should support the reliability of the prognostic signature in glioma patient OS prediction. Further, we downloaded the expression profiles of The Cancer Genome Atlas GBM project along with their clinical information and tested the performance of the risk score in predicting their OS. As a result, a total of 153 glioma patients out of 617 samples were found to be with expression value and complete clinical information. As expected, the 76 samples with higher risk score had poorer prognosis than those with lower risk score (*P* = 0.043, hazard ratio (HR) = 0.832, Fig. S1).

To test whether differences exist in patients’ clinical and pathological characteristics between the high‐risk and low‐risk groups, we compared their age, gender, treatment and *O* ^6^‐methylguanin‐DNA methyltransferase (MGMT) status. None of these characteristics was significantly different (age: *P* = 0.731, *t*‐test; gender: *P* = 1; treatment: *P* = 0.627; MGMT status: *P* = 0.311, chi‐square test). With multivariate analysis when taking age, gender, treatment and MGMT status into consideration along with risk score, treatment was shown to have a significant influence on the risk score prediction power (*P* = 2.0 × 10^−6^). Therefore it would be better to take the treatment status or method into consideration when available in glioma OS prediction.

### Gene set enrichment analysis

**Figure 5:**
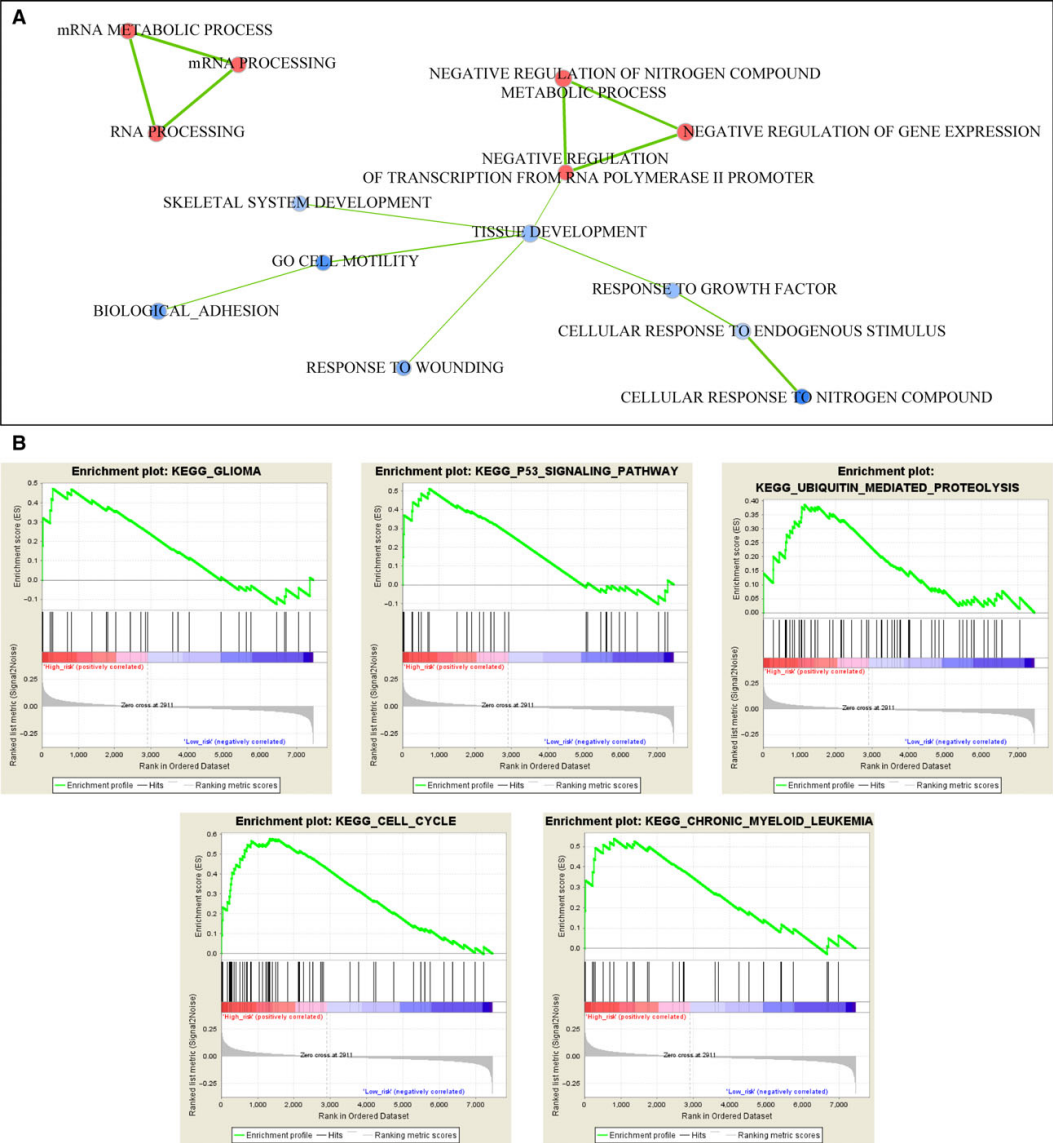
Gene set enrichment analysis of gene expression profiles in the training set with samples stratified by their risk scores. (A) Visualization of significantly enriched biological process terms that grouped in terms of their shared genes. Blue and red node indicates down‐ and up‐regulated terms in samples with high risk score, respectively, and edge indicates at least one shared gene between two terms. (B) Significantly up‐regulated KEGG pathways in glioma samples with high risk score.

Glioma samples in the training set were grouped by their risk score by using median risk score as the cut‐off, and their gene expression profiles were imported into gsea software for screening biological processes and pathways associated with the prognostic signature. Figure 5A illustrates the visualization of significantly enriched biological processes that grouped by their related genes. Significantly up‐regulated pathways in samples with high risk score are shown in Fig. 5B. Significant enrichment of cancer‐related processes or pathways could be observed, such as p53 signaling pathway, cell mortality, cell cycle and so on.

### Expression of *LOC441179*,* PON2* and *USP46‐AS1* in glioma cells

**Figure 6:**
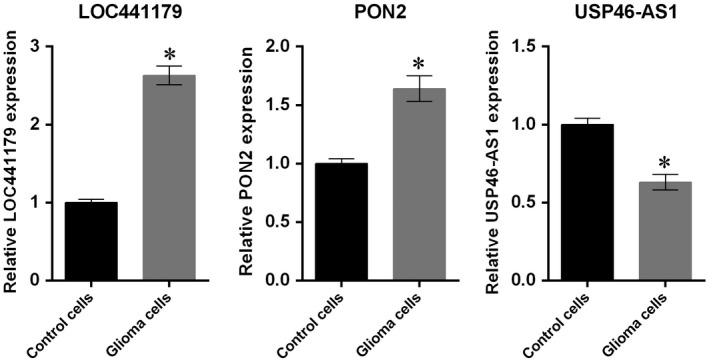
Expression of *LOC441179*,*PON2* and *USP46‐AS1* in glioma cells compared to control cells. Data are expressed as mean ± SD from three replicates. Student's *t* test was used to compare their relative mRNA level between glioma and control cells; *P < 0.05.

qRT‐PCR was performed to investigate the expression of *LOC441179*,* PON2* and *USP46‐AS1* in glioma cells compared to control cells. As shown in the Fig. 6, the relative expression of *LOC441179* and *PON2* in glioma cells was significantly higher than in control cells (*P *<* *0.05). However, the relative expression of *USP46‐AS1* in glioma cells was markedly decreased compared to the control cells (*P *<* *0.05).

## Discussion

As the most frequent primary malignant tumor in the CNS, glioma causes abundant mortality in children and adults throughout the world. Traditional surgical treatment and chemoradiotherapy are difficult to conduct because of diffuse brain infiltration 37, and early diagnosis is also difficult to achieve. In this study, we analyzed ncRNA expression profiles of 80 glioma samples from GEO and identified a ncRNA‐based prognostic signature composed of the weighted expressions of *USP46‐AS1*,* PON2* and *LOC441179*; based on this, glioma samples could be divided into high‐ and low‐risk groups with significantly different OS. Kaplan–Meier analysis indicated that the patients with lower expression of *LOC441179* and *PON2* had a higher survival rate than patients with higher expression of *LOC441179* and *PON2*; on the contrary, the down‐regulated expression of *USP46‐AS1* was significantly associated with a higher survival rate. Paraoxonase 2 (PON2) is an intracellular protein that belongs to the endogenous free‐radical scavenging enzyme system 38. In agreement with our finding, overexpression of PON2 is observed in solid cancers derived from liver, prostate, kidney, pancreas and thymus 39. The glioma cell line GBM expressed a higher PON2 protein level compared with normal brain tissue, providing support for the importance of PON2 for cancer cell survival. Ubiquitin specific peptidase 46 (USP46) belongs to a large family of cysteine proteases that function as deubiquitinating enzymes and are highly expressed in brain 40.

Survival rate is an important indicator of prognosis after treatment, and the significant association of survival rate with the expression levels of *USP46‐AS1*,* PON2* and *LOC441179* suggests that those three ncRNAs should be important indicators for prognosis prediction for glioma patients. Functional studies of ncRNAs are comparatively scant. Previous studies have demonstrated that prognostic ncRNAs tend to be clustered significantly in immune‐related GO biological processes and four KEGG biological pathways 41. Therefore, we performed a comprehensive functional study for the feasibility of diagnostic or prognostic roles of ncRNAs in gliomas.

Through GO enrichment analysis of differentially expressed ncRNAs, three related physiological processes were obtained, involving mRNA processing, gene expression regulation, and cell adhesion and stress response. Up‐regulation of ncRNAs was frequently observed in the mRNA processing and gene expression regulation processes, which indicated that many ncRNAs may act as post‐transcriptional regulators participating in mRNA processing and protein expression processes, such as pre‐mRNA splicing, mRNA turnover and mRNA translation in glioma 42. Cell adhesion is an important process in tumor cell migration and invasion, and decreased cell adhesion promotes tumor cell migration. During invasion, cell adhesion is necessary for tumor cells to interact with the extracellular matrix 43, 44. Down‐regulated ncRNAs in the process of cell adhesion may affect the cell adhesive force, and thereby promote the invasion and migration of glioma cells.

KEGG pathway enrichment analysis of the differentially expressed ncRNAs was conducted in the high‐ and low‐risk groups, and five significantly enriched pathways were obtained in the high‐risk group, including glioma, the p53 signaling pathway, ubiquitin‐mediated proteolysis, cell cycle and chronic myeloid leukemia. The p53 signaling pathway plays an important role in tumorigenesis. Lin *et al*. 45 reported that p53 expression was positively correlated with brain glioma, and an elevated expression of p53 was observed in high‐grade compared with low‐grade glioma. A meta‐analysis of the expression and prognostic significance of glioma patients conducted by Jin *et al*. 46 reported that p53 positively correlated with the glioma grades and significantly associated with 1‐, 3‐ and 5‐year OS. This suggested a tight correlation of the p53 signaling pathway with the grading and prognosis of glioma. The ubiquitin–proteasome system functions in the degradation of damaged proteins in cells 47. Epidermal growth factor receptor (EGFR) gene mutations are positively correlated with glioblastoma, and Schmidt *et al*. 48 reported that ubiquitin‐mediated protease degradation of mutant EGFR prevented the recycling of EGFR back to the cell surface. Eukaryotic elongation factor‐2 (eEF‐2) kinase was found to be overexpressed in glioma cells and could promote protein translation and cell proliferation, and the ubiquitin–proteasome system could regulate the critical expression of genes for cell survival and proliferation by eEF‐2 kinase degradation 49. The cell cycle is tightly regulated by various signaling pathways in mammalian cells, and disorder of the cell cycle leads to the continuous activation of cell proliferation in many malignant tumors, included glioma 50. Abundant studies showed that miRNAs could regulate the cell cycle through interacting with the target genes in glioma. Dong *et al*. reported that overexpressed miR‐21 promoted the tumor progression of gliomas through inhibiting the expression of p53 in tumor cells, and down‐regulated miR‐21 led to proliferation repression and cell cycle arrest in glioma cells 50, 51. In addition, miRNA‐128 could influence the stability of the cyclin‐dependent kinase 1–cyclin B complex by targeting protein WEE1 to regulate the G2/M transition in malignant glioma 52, 53. KEGG pathway analysis also indicated the association of chronic myeloid leukemia and glioma, and this was in accordance with previous research showing that somatic non‐synonymous coding mutations in patients with myeloid cell leukemia‐1 could accelerate the progression of glioma by enhancing the stability of tumor cells 54.

In summary, as glioma is the most prevalent tumor in the CNS, exploration of new diagnostic methods and useful biomarkers is urgently needed. In this study, we explored the association between ncRNA expression and glioma OS, and obtained a prognostic signature containing three ncRNAs, including *USP46‐AS1*,* PON2* and *LOC441179*, based on which we could accurately separate glioma patients with better prognosis from those with a worse prognosis. This should be helpful for the selection of suitable treatments and improvement in prognosis of glioma patients.

## Author contributions

JX and YF conceived and designed the project, QZ acquired the data, JX and XL analyzed and interpreted the data, XG and XL wrote the paper. JX and YF approved the final version.

## Conflict of interests

The authors declare no conflict of interest.

## Supporting information


**Fig. S1.** Overall survival curves of glioma patients from The Cancer Genome Atlas with higher (red curve) and lower (green curve) risk score. Kaplan–Meier survival analysis along with log‐rank test was applied for comparing overall survival between the two glioma groups.
