# Supplementary figures and images for: A prognostic signature based on three non‐coding RNAs for prediction of the overall survival of glioma patients
Source: FEBS Open Bio. 2019 Mar 7;9(4):682–92. doi: 10.1002/2211-5463.12602 (PMC6443874; doi:10.1002/2211-5463.12602)

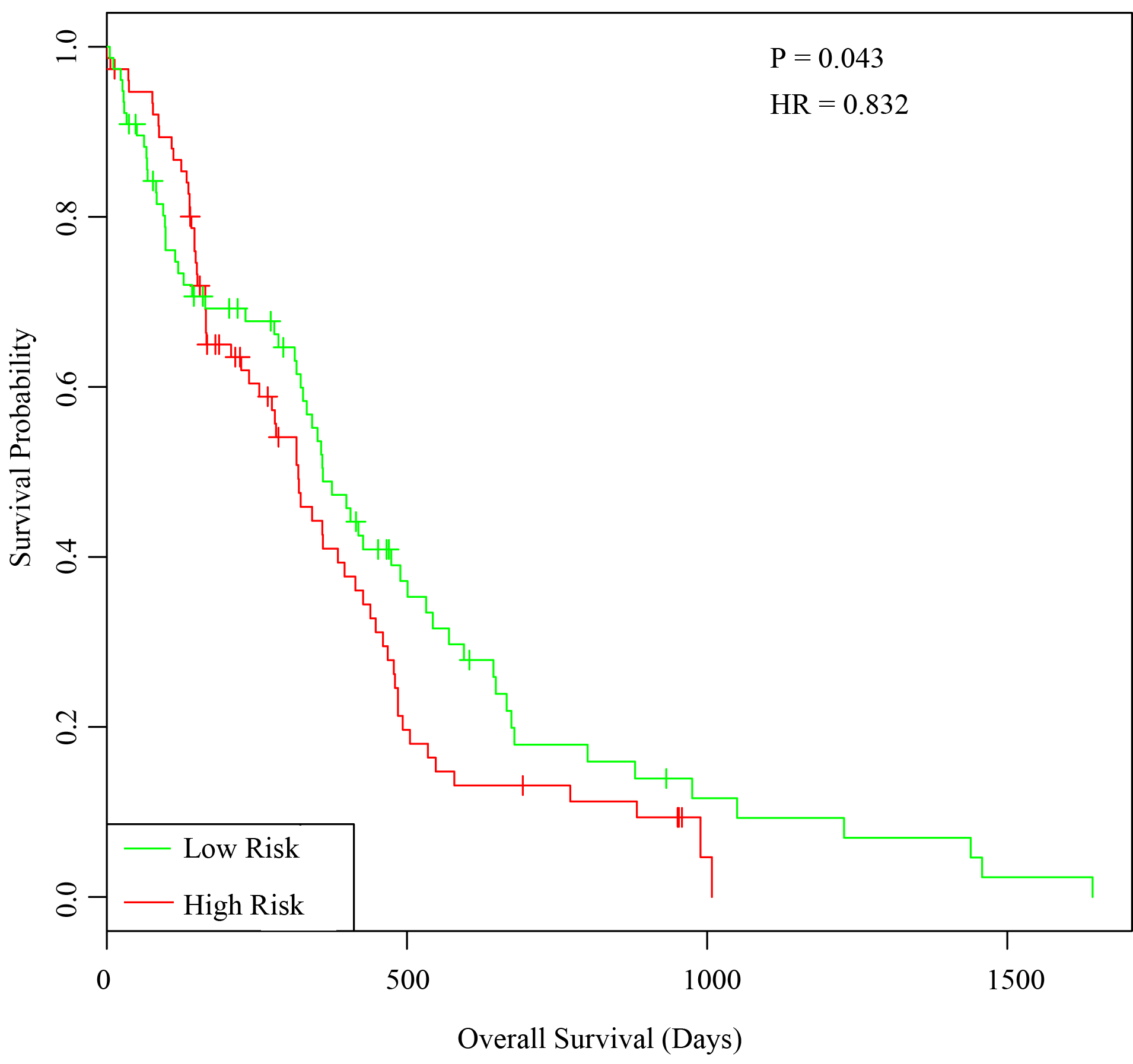

Supplement: Supplementary file 1 — Fig. S1. Overall survival curves of glioma patients from The Cancer Genome Atlas with higher (red curve) and lower (green curve) risk score. Kaplan–Meier survival analysis along with log‐rank test was applied for comparing overall survival between the two glioma groups. [file FEB4-9-682-s001.tif]
